# The Interaction Between Lentiviral Integrase and LEDGF: Structural and Functional Insights

**DOI:** 10.3390/v1030780

**Published:** 2009-11-06

**Authors:** Stephen Hare, Peter Cherepanov

**Affiliations:** Division of Medicine, Imperial College London, St. Mary’s Campus, Norfolk Place, London, W2 1PG, UK; E-mail: s.hare@imperial.ac.uk

**Keywords:** retrovirus, integrase, host factors, protein-protein interaction

## Abstract

Since its initial description as an HIV-1 integrase (IN) interactor seven years ago, LEDGF has become one of the best-characterized host factors involved in viral replication. Results of intensive studies in several laboratories indicated that the protein serves as a targeting factor for the lentiviral DNA integration machinery, and accounts for the characteristic preference of *Lentivirus* to integrate within active transcription units. The IN-LEDGF interaction has been put forward as a promising target for antiretroviral drug development and as a potential tool to improve safety of lentiviral vectors for use in gene therapy. Additionally, as a natural ligand of lentiviral IN proteins, LEDGF has been successfully used in structural biology studies of retroviral DNA integration. This review focuses on the structural aspects of the IN-LEDGF interaction and their functional consequences.

## 1. Introduction

**Figure 1.**
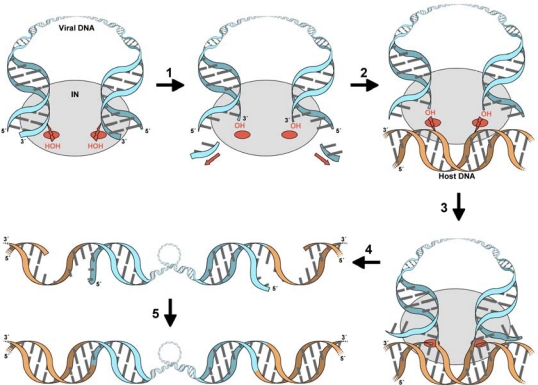
The retroviral DNA integration pathway. The PIC, formed following reverse transcription of the viral RNA genome in the host cell cytoplasm, contains viral DNA (light blue), IN (grey oval), along with other viral and host cell proteins (not shown). Within the PIC, the active sites of IN (red ovals) activate water molecules for nucleophilic attacks on the phosphodiester backbone close to the 3′ ends of the viral DNA. This 3′-processing reaction (1) results in the removal of a di- or tri-nucleotide from both 3′ ends of the viral DNA, exposing the reactive 3′ hydroxyl groups attached to invariant CA dinucleotides. Following nuclear import, the PIC comes into contact with host chromosomal DNA (orange) (2). Whereupon, the IN active sites activate the hydroxyl groups at the 3′ viral DNA ends to cut a pair of phosphodiester bonds in the opposing strands of chromosomal DNA, 4–6 bp apart (the exact separation depends on the retroviral genus, and equals 5 bp for lentiviruses) (3). The resulting intermediate (4) contains viral DNA joined at each 3′ end to chromosomal DNA, flanked by short gaps and 5′-overhangs. The final DNA repair step (5) that joins the 5′ viral DNA ends to the host DNA is presumably carried out by host proteins.

For successful replication, HIV and other retroviruses depend on virally encoded IN enzymes to orchestrate insertion of their reverse transcribed genomes into host cell DNA (reviewed in [1,2]). The active site of retroviral IN catalyzes two distinct nucleophilic substitution (SN2) reactions during the integration process (Figure 1). Firstly, the 3′ processing reaction takes place in the cytoplasm of the host cell in the context of a large nucleoprotein complex, termed the preintegration complex (PIC). In this step, IN removes two or three nucleotides from the 3′ ends of the viral DNA, exposing the reactive 3′ hydroxyl groups of the invariant CA dinucleotides. The second reaction, strand transfer, occurs in the nucleus and involves a pair of coordinated transesterification reactions that cut both strands of target DNA, simultaneously joining them to the 3′-ends of the viral DNA molecule. These concerted strand transfer events target a pair of phosphodiester bonds on the opposing strands of the target DNA, across its major groove. Consequently, following gap repair by host enzymes, the resulting provirus is flanked by short (4–6 bp, depending on the retroviral genus) duplications of the target DNA sequences.

Retroviral IN comprises three domains, an N-terminal domain (NTD) containing the invariant Zn^2+^-binding HHCC motif, a catalytic core domain (CCD) containing the active site, and a positively charged C-terminal domain (CTD) [3–6]. The IN active site contains three invariant acidic residues, forming the so-called D,DX_35_E motif [6,7]. Based on analogy with distantly related polynucleotidyl transferases, most notably prokaryotic transposases and ribonuclease H, the IN catalytic triad carboxylates are expected to coordinate a pair of Mg^2+^ cations [8–12]. All three IN domains have been implicated in multimerization [13–16] and DNA binding [3,17–19]. In particular, the CCD of HIV-1 IN was shown to participate in sequence-specific recognition of viral DNA termini [20,21]. Solution NMR and X-ray crystallography have been used to determine the structures of the individual domains and two-domain fragments of retroviral INs [22–30] (reviewed in [31]). While each isolated domain is dimeric in solution, recombinant full-length retroviral INs exist in varying multimeric states and, with only rare exceptions [30], are highly prone to aggregation. The two active sites of the spherical CCD dimer are located on opposing faces, separated by ∼40 Å [22]. Therefore, it follows that a tetramer of IN would be the minimal protomer to correctly position a pair of active sites for concerted strand transfer events targeting phosphodiester bonds across the major groove (∼18 Å). A growing number of recent reports suggest that the tetrameric form of IN is indeed its functionally-relevant state [16,32–37].

Given a historical misnomer, lens epithelium derived growth factor (LEDGF) is a ubiquitous chromatin-associated protein with poorly characterized cellular functions. LEDGF is most notorious for its tight interaction with lentiviral INs and its role in HIV-1 replication. First implicated in virology as a cellular binding partner of ectopically expressed HIV-1 IN, LEDGF was also found to stimulate its enzymatic activity *in vitro* [38]. The protein-protein interaction was soon corroborated by two independent laboratories [39,40]. The functional aspects of this virus-host interaction were recently reviewed [41,42]; herein, we primarily focus on its structural details.

## 2. Domain organization of LEDGF

LEDGF belongs to the hepatoma derived growth factor (HDGF) related protein (HRP) family and is by far the most extensively studied IN binding partner. Predicted to be largely disordered, LEDGF contains two small structural domains [43]. One of these, the PWWP domain (LEDGF residues 1–91) is present at the N-termini of all HRP family members [43–46]. Together with the nuclear localization signal (residues 148–156) and a pair of AT-hook motifs (residues 178–197), the PWWP domain is responsible for the tight association of LEDGF with chromatin [47–50]. The second structural domain is located within the C-terminal region of LEDGF (residues 347–429). Found responsible for the interaction with lentiviral INs, it was termed the integrase-binding domain (IBD) [43,48]. An alternative splice form of LEDGF, p52, lacks the IBD and concordantly fails to interact with HIV-1 IN or activate its enzymatic activity [43,51]. Of the five other human HRP family members, only HRP2 contains a conserved IBD within its C-terminal region, enabling it to interact with and stimulate the strand transfer activity of HIV-1 IN *in vitro* [43]. However, it is important to note that the affinity of HIV-1 IN for HRP2 is markedly lower than it is for LEDGF [43]; it is currently unclear whether HRP2 has any function in lentiviral replication.

The IBD was reported to mediate interactions between LEDGF and a number of cellular proteins: JPO2, a putative transcription factor [52,53]; Menin:MLL, a histone methyltransferase complex involved in transcriptional regulation and oncogenesis [54]; and PogZ, a protein of yet unknown function [55]. Intriguingly, JPO2 and Menin:MLL were shown to be tethered to chromatin by LEDGF [52,54]. Based on its domain organization, intracellular localization and characterized cellular binding partners, LEDGF is likely to play a role in regulation of gene expression, and/or as an adaptor protein tethering a plethora of cellular proteins to chromatin.

## 3. The role of LEDGF in lentiviral infection

The initial insight into possible role(s) of LEDGF in lentiviral replication came from early experiments that used ectopically-expressed IN. Unexpectedly, the chromatin binding activity of HIV-1 IN, initially thought to be intrinsic to this protein [56], was shown to be dependent on endogenous LEDGF [51,57]. Additionally, stability and nuclear accumulation of HIV-1 IN in human cells were drastically impaired by LEDGF depletion [51,57,58]. These results suggested that LEDGF might tether IN to host cell chromatin and that it may also be involved in its nuclear import and protection from proteasomal degradation. The early steps of viral infection are essentially single-molecule events and thus present substantial challenges to studies of ubiquitous host factors. Consequently, it required considerable efforts to generate cell lines with sufficient levels of LEDGF depletion or genetic knockout models to arrive at a consensus on the importance of this protein for lentiviral DNA integration. HIV-1 infection of cells depleted for or lacking LEDGF was substantially reduced relative to controls, due to a specific block at the integration step [59–61]. The infectivity could be restored by re-expression of full-length LEDGF, while over-expression of the isolated IBD lead to even more drastic suppression of HIV-1 integration [60,62]. So far no evidence has emerged to support a role of LEDGF in nuclear import or protection of the lentiviral PIC, although HIV-1 and feline immunodeficiency virus (FIV) PICs can be immunoprecipitated with anti-LEDGF antibodies [57].

The interaction with LEDGF is exclusive to INs from the retroviral genus of *Lentivirus*, while those from the members of *Alpha*-, *Beta*-, *Gamma*-, *Deltaretrovirus*, and *Spumavirus* genera do not bind LEDGF [57,63,64]. The characteristic features of lentiviruses include their marked propensity to integrate within active transcription units of the host cell genome, and their bias against insertion into promoters and CpG islands [65–70] (reviewed in [71]). Using LEDGF knockdown and knockout models, these properties were shown to depend on the IN-LEDGF interaction [61,72,73]. Intriguingly, with respect to genomic features, the integration site profiles of HIV-1 in the absence of LEDGF are reminiscent of non-lentiviral genera [61,73].

**Figure 2.**
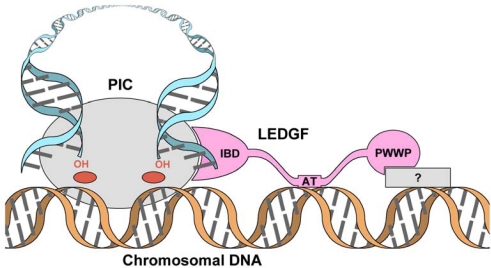
The role of LEDGF in lentiviral biology. Representations of IN and viral and host DNA are conserved from Figure 1. LEDGF (pink) interacts with the PIC via its C-terminal IBD, with host DNA via its AT-hooks and with an unidentified component of the chromatin (grey rectangle) via its N-terminal PWWP domain, tethering the PIC to select loci of host cell chromatin.

The current model for LEDGF function in lentiviral integration advocates that the N-terminal region of LEDGF interacts with the host chromatin at active transcription units and, via the connected IBD, brings the PIC into proximity with this preferred region of the genome for integration while stimulating IN strand transfer activity (Figure 2). Presumably, integration into transcriptionally-active genomic loci improves the efficiency of lentiviral gene expression, providing a sufficient evolutionary advantage [74].

This model suggests an exciting possibility for engineering artificial LEDGF-like molecules for targeting integration of lentiviral gene therapy vectors. The concern over using integrating vectors is a tangible danger of insertional mutagenesis. Indeed, several unfortunate cases of leukemia associated with gammaretroviral vector insertions in the vicinity of a proto oncogene have been observed in two gene therapy trials [75,76]. An artificial targeting factor linking the IBD of LEDGF to a DNA binding domain, able to selectively associate with a safe genomic locus, could in principle be used to direct lentiviral integration. The potential for this was demonstrated *in vitro* using a fusion construct comprising the IBD of LEDGF and the DNA binding domain of the λ phage repressor. The chimeric construct targeted a modest fraction of IN strand transfer events into the vicinity of λ repressor binding sites [77]. More recently, further validation of this approach was provided by Meehan *et al*. [78] who fused heterologous chromatin binding domains to the IBD and showed that the chimeras were able to rescue both IN chromatin tethering and HIV-1 integration under conditions of endogenous LEDGF depletion.

## 4. The primary IN:LEDGF interface

**Figure 3.**
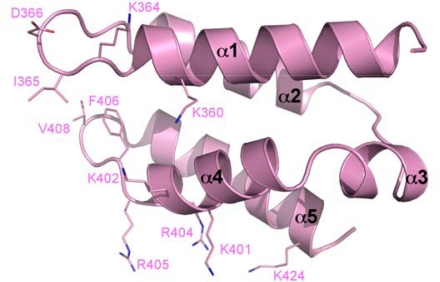
The solution structure of the LEDGF IBD. The helical bundle is shown in cartoon representation, with individual helices labeled (PDB ID 1z9e). Side chains contributing to the hydrophobic area at the left side of the helical bundle as drawn and the positive face on the underside are shown as sticks and labeled.

The solution structure of isolated LEDGF_IBD_ was determined by NMR spectroscopy (PDB ID 1z9e), revealing a bundle of four long α-helices (α1, α2, α4, and α5) (Figure 3) [79]. At one end of the bundle, hairpin turns connect α1 to α2 and α4 to α5. Meanwhile, at the other end of the structure, a shorter α-helix (α3) links α2 and α4. Several exposed hydrophobic side chains are located on the two adjacent hairpin turns and alanine scanning mutagenesis revealed three of these residues (Ile-365, Phe-406 and to a lesser extent Val-408) to be involved in the interaction with HIV-1 IN. The adjacent acidic residue Asp-366 was shown by mutation to Asn (D366N) to be essential for both the interaction with IN and for stimulating its enzymatic activity [79]. This loss-of-function mutation has since been widely used in studies of the roles of LEDGF in HIV-1 replication. Fortuitously, mutations of Asp-366 do not seem to affect the interaction of LEDGF with its known cellular binding partners [52,53,55].

**Figure 4.**
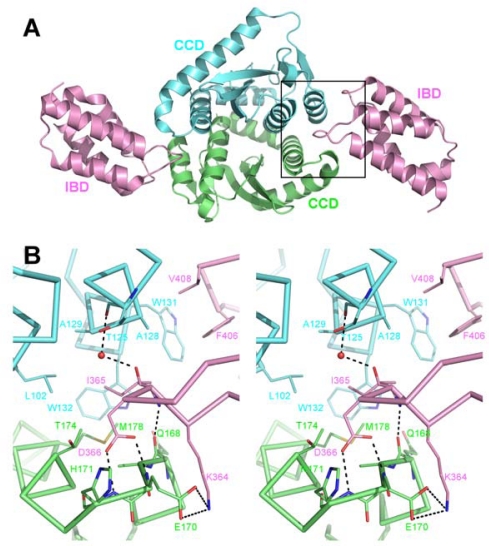
The primary IN:LEDGF interface. **A.** Cartoon representation of the co-crystal structure of the IN_CCD_:LEDGF_IBD_ complex (PDB ID 2b4j). IN chains are colored green (chain A) and cyan (chain B) and a pair of LEDGF chains interacting at either end of the IN CCD dimer are pink. **B.** Stereo close-up view of the region enclosed by a black rectangle in **A**, showing details of the CCD:IBD interface. The protein backbone and side chains, shown in ribbon and stick representations, respectively, are colored by atom. Side chains of residues involved in interactions are shown, as well as a water molecule coordinated between main chain carbonyls of IN Thr-125 and LEDGF Ile-365.

The primary site of LEDGF interaction with IN is the CCD and the details of this interface were revealed in a co-crystal structure containing HIV-1 IN_CCD_ and LEDGF_IBD_ constructs (Figure 4) (PDB ID 2b4j) [80]. The loop connecting α1 and α2 of the IBD inserts into the CCD dimer interface and interacts with both monomers of the CCD dimer. Here, the side chain of Asp-366 makes dual hydrogen bonds with the main chain amino groups of IN chain A residues Glu-170 and His-171, located on the loop connecting CCD alpha helices α4 and α5 (known as α4/5 connector). The hydrophobic side chain of LEDGF Ile-365 makes Van der Waals interactions with the side chains of IN chain A residue Met-178 and chain B residues Leu-102, Ala-128, Ala-129, Trp-131 and Trp-132 (Figure 4B). The second IBD loop at this location contains the hydrophobic side chains of Phe-406 and Val-408, which pack against a hydrophobic patch on IN chain B formed by Ala-128 and Trp-131 (Figure 4B).

As well as identifying LEDGF residues crucial for the interaction with IN, mutational analyses highlighted HIV-1 IN Val-165, Arg-166, Gln-168, Leu-172, and Lys-173 as being important for the interaction [39,79,81]. Interestingly, these residues do not form direct interactions with LEDGF. Mutations at these positions are thought to affect conformation of the α4/5 connector, disrupting the surface complementarity between the CCD and the IBD [80]. Side chains of HIV-1 IN residues Ala-128, Ala-129, Trp-131 and Trp-132 directly interact with the IBD and were also found to have varying effects on the strength of the HIV-1 IN-LEDGF interaction [80–82]. Mutations within or close to the α4/5 connector typically result in lethal viral phenotypes, initially attributed to a defect in nuclear import (V165A and R166A, [83]) or their inability to interact with LEDGF (Q168A, [39]). However, follow up analyses revealed a more complex picture with the mutant viruses displaying additional defects in reverse transcription [82,84,85], which cannot be explained merely by disruption of the virus-host interaction [60,61].

Intuitively, this primary IN-LEDGF interaction is of interest for the development of a novel class of antiretrovirals. The LEDGF binding cavity on the IN CCD dimer interface has the potential for both hydrogen bonding and hydrophobic interactions, suggesting a small molecule could specifically bind there with high affinity. A number of ligands have been observed binding there in crystal structures of IN fragments ([86]; S.H. and P.C. unpublished). Chemical library screening approaches and *in silico* pharmacophore modeling have been used to identify lead inhibitors of HIV-1 IN-LEDGF interaction [87–89].

Recent structures of LEDGF_IBD_ bound to INs of HIV-2 and maedi-visna virus (MVV) have revealed the conservation of the co-factor binding at this same pocket [16,90]. Retroviruses are notorious for their high evolutionary rate; hence conservation of this interaction throughout the entire genus indicates its vital importance for lentiviral biology. HIV-2 and MVV INs share approximately 60% and 30% amino acid sequence identity with HIV-1 IN, respectively. While HIV-2 IN binds the IBD in a manner nearly identical to that of HIV-1 IN, the IBD shows a twist of ∼34° when binding to the more divergent MVV IN. This is due to a slight change in the size and shape of the MVV CCD pocket, a result of local amino acid changes [16]. More drastic differences in the local structures of the analogous pockets at the CCD dimer interfaces of non-lentiviral INs (in particular the conformations of their α4/5 connectors) explain the notable lentiviral specificity of LEDGF [30,80].

## 5. The IN NTD and the high affinity IN-LEDGF interaction

**Figure 5.**
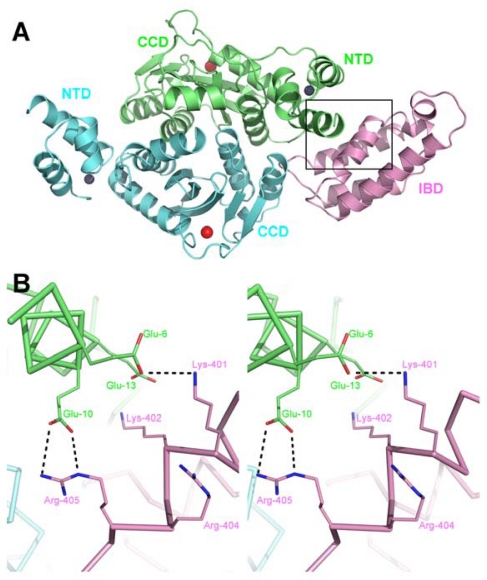
The interaction between the IBD and IN NTD. **A.** Cartoon representation of the HIV-2 IN_NTD+CCD_ and LEDGF_IBD_ co-crystal structure (PDB ID 3f9k) with the IN dimer colored green (chain A) and cyan (chain B) and LEDGF colored pink. Red spheres represent magnesium ions in the IN active sites and dark grey spheres represent Zn^2+^ ions coordinated by the HHCC motif of the NTDs. **B.** Details of the NTD:IBD interface, showing the area enclosed by a black rectangle in **A**. The charge-charge interactions are shown as black dashed lines between the stick representations of the side chains involved.

Although the CCD of HIV-1 IN is essential and minimally sufficient for the interaction with LEDGF, the NTD is required for high-affinity binding [51]. Thus, the HIV-1 IN H12N mutant, with disrupted NTD structure, was unable to interact with LEDGF in pull-down assays and required over-expression of LEDGF to associate with condensed chromatin in mitotic cells [51]. The crystal structure of LEDGF_IBD_ in complex with a two-domain construct of HIV-2 IN containing its NTD and CCD revealed the details of this interaction (PDB ID 3f9k) (Figure 5) [90]. In this structure, while the previously identified IBD:CCD interaction is preserved, the positive face of the IBD makes additional contacts with a negatively charged surface of the NTD. Specifically, IBD residues Lys-401, Lys-402, Arg-404, and Arg-405 oppose HIV-2 IN Glu-6, Glu-10, and Glu-13 (Figure 5B). Pull-down and yeast two-hybrid experiments using mutants targeting this interface confirmed its importance for the protein-protein interaction. Additionally, it was observed that reversing the charges on both sides, *i.e*. making Lys/Arg to Glu mutations on LEDGF and Glu to Lys substitutions on IN, recuperated the interaction. *In vitro* concerted integration assays and single round HIV-1 infection experiments using reverse charge mutants showed the cofactor role of LEDGF also depends on this interface. These activity and infection assays also indicated that the charge-charge interface could be reversed, with mutant INs requiring LEDGF containing complementary mutations for activity. Howbeit, the reversed mutant combinations were significantly less active/infectious than the wild type [90].

## 6. A role for LEDGF in IN tetramerization

Structural information detailing protein-protein interfaces involved in PIC assembly and the organization of the IN active site is invaluable for the development of antiretroviral drugs. The odds of obtaining useful crystals can often be improved by using a natural or even artificial ligand for the target protein. Typically, complexes of lentiviral INs with full-length LEDGF or LEDGF_IBD_ are considerably more soluble than their free forms (unpublished observations). Furthermore, LEDGF can be expected to stabilize the functionally-relevant conformation(s) of divergent lentiviral INs. Crystallization and structure determination of two such complexes containing a two-domain fragments of HIV-2 and MVV IN_NTD+CCD_ helped to elucidate the mechanism of functional IN tetramerization [16,90]. Notably, while crystallization of unliganded HIV-1 IN fragments required presence of various solubilizing point mutations [22,25,26,91], the analogous changes were not necessary to obtain crystals of HIV-2 or MVV IN_NTD+CCD_ complexed with LEDGF_IBD_ [16,90].

**Figure 6.**
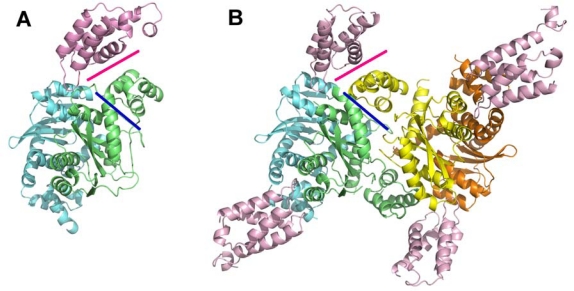
The structural basis for IN tetramerization. **A.** The crystal structure of the dimeric HIV-2 IN_NTD+CCD_ construct in complex with LEDGF_IBD_ (PDB ID 3f9k). The intramolecular NTD:CCD interface is shown as a blue line and the IBD:NTD interface as a pink line. **B.** The crystal structure of tetrameric MVV IN_NTD+CCD_ in complex with LEDGF_IBD_ (PDB ID 3hph). The NTD-CCD interface (blue line) is conserved from the dimeric HIV-2 structure, although, as a consequence of NTD swapping, in the tetrameric structure this interface is intermolecular. Charge-charge interactions between the IN NTD and LEDGF IBD (pink line) act to stabilize the tetramer thereby explaining the observed effect of LEDGF on IN multimerization *in vitro* [16,36].

The co-crystal structures of MVV IN_NTD+CCD_ with LEDGF_IBD_ (PDB IDs 3hpg and 3hph) revealed a series of IN tetrameric arrangements [16], while a similar HIV-2 - derived complex (PDB ID 3f9k) was captured in a dimeric form [90]. Importantly, the dimer-of-dimers tetrameric architecture observed in the crystals of the MVV complex is very similar to that reported earlier for the unliganded HIV-1 IN_NTD+CCD_ construct (PDB ID 1k6y) [26], despite less than 30% amino acid sequence identity between MVV and HIV-1 INs. The IN dimer-dimer interface is stabilized by an NTD of one dimer interacting with a CCD of the opposing dimer. The analogous NTD:CCD interface is also observed in the crystals of dimeric HIV-2 IN_NTD+CCD_:LEDGF_IBD_, although in this case the NTD interacts with its own CCD dimer [90]. These observations indicated the mechanism for IN tetramerization that involves swapping of a pair of NTDs between interacting dimers. Disruption of the NTD:CCD interface abrogates tetramerization and dramatically reduces the enzymatic activities of HIV-1 IN [16,36,92]. The long and flexible NTD-CCD linkers allow striking flexibility of the dimer-dimer IN interface within the two-domain IN constructs [16]. Based on the tetrameric IN_NTD+CCD_ structures, the LEDGF binding platform includes the CCDs from one IN dimer and an NTD from another (Figure 6). Concordantly, LEDGF binding dramatically stimulates tetramerization of HIV-1 IN *in vitro* [16,36]. Furthermore, the co-factor can partially rescue multimerization of HIV-1 IN mutants with defects in the NTD:CCD interface [16]. Intriguingly, Hayouka *et al*. [93], reported that peptides derived from the LEDGF IBD loops (residues 361–370 and 402–411) promoted HIV-1 IN multimerization *in vitro*. This observation suggests that engagement of the primary IBD:CCD interface alone, possibly through allosteric effects on the crucial CCD:NTD interface, could stabilize the tetramer.

The stoichiometry of the biologically-relevant IN:LEDGF complex is yet to be resolved. The crystal structures of the MVV IN_NTD+CCD_:LEDGF_IBD_ complexes contained four LEDGF chains associated with each IN tetramer, forming both primary and secondary interfaces with IN [16]. This same 1:1 stoichiometry was observed earlier in crystals of the minimal HIV-1 IN_CCD_:LEDGF_IBD_ complex [80]. However, HIV-2 IN_NTD+CCD_ construct was co-crystallized with a single LEDGF chain per IN dimer [90], which could also be attributable to crystal packing forces. Therefore, the available structural data therefore support either 1:1 or 2:1 IN:LEDGF stoichiometry. Mass spectrometry analyses of full-length HIV-1 IN:LEDGF complex produced by co-expression in bacteria [37] and size exclusion chromatography of IN:LEDGF_IBD_ complexes assembled *in vitro* [36] argued for the latter stoichiometry, although more data are required to ascertain its biological relevance. While it is reasonable to speculate that the functional IN tetramer might possess as many as four high affinity binding sites for LEDGF, as the main proposed role of the host factor is tethering the PIC to chromatin, a single LEDGF molecule might well suffice.

## 7. Effects of LEDGF on enzymatic activities of lentiviral IN

Retroviral DNA integration can be re-constituted *in vitro* using recombinant IN, viral DNA mimics (commonly referred to as donor DNA) and target DNA [94,95]. Typically, such reactions lead to formation of abundant Y-shaped strand transfer products, resulting from the insertion of a single donor DNA end into one strand of a target DNA molecule. Under optimized conditions, it is possible to observe biologically-relevant concerted strand transfer products, arising from coordinated insertions of pairs of donor DNA molecules. The IN tetramer has been implicated as the basic catalytic unit for both 3′-processing and strand transfer reactions [16,32,96]. While residual 3′-processing and half-site integration can be carried out by IN mutants impaired for tetramerization, the tetramer is essential for concerted integration [16]. Accordingly, HIV-1 IN tetramers were observed within *in vitro* assembled nucleoprotein complexes competent for concerted integration [34].

In accordance with its role in lentiviral IN tetramerization, LEDGF robustly stimulates its 3′-processing and strand transfer activities *in vitro* [36,38,64,90,97–99]. Nevertheless, ambiguity persisted over the effect of LEDGF on concerted HIV-1 integration. Cryptically, depending on reaction conditions such as the order of addition and relative input ratios of HIV-1 IN and LEDGF, the type of DNA substrates, and the reaction buffer components, the co-factor can both stimulate and inhibit concerted HIV-1 integration *in vitro* [36,90,98,99]. Thus, Raghavendra and Engelman [99] observed that while LEDGF promoted overall levels of strand transfer activity of HIV-1 IN, it specifically inhibited formation of concerted integration products. Pandey *et al*. [98] went further and showed that, under similar conditions, while using excess LEDGF inhibited concerted integration, a modest (two- to three- fold) stimulation was observed when using equimolar or lower ratios of LEDGF to HIV-1 IN, results that were later replicated by an independent group [36]. Pandey *et al*. [98] also showed that, in order to observe the simulative effect of LEDGF on HIV-1 concerted integration, it is important to add donor DNA substrate before LEDGF, suggesting that the host factor might prevent IN from forming a productive complex with donor DNA [98]. As LEDGF binding locks HIV-1 IN into a tetrameric state [36,93], perhaps there is insufficient flexibility to subsequently engage a pair of viral DNA ends. During infection this is unlikely to be an issue, as PIC assembly occurs in the cytoplasm, and LEDGF may not be encountered prior to nuclear entry. Consistent with this view, PIC assembly proceeds normally in LEDGF-null cells, and therefore does not depend on LEDGF [61]. More recently it was shown that in the presence of LEDGF and higher inputs of donor DNA, HIV-1 IN displays very robust concerted strand transfer activity, albeit significant levels of half-site strand transfer persist [90]. Under these conditions, the NTD:IBD interface was specifically important for stimulation of the concerted strand transfer activity of HIV-1 IN. It is important to note that the relative ratio of concerted to half-site strand transfer products greatly depends on the viral source of the IN used. For example, while HIV-1 IN even under most optimized conditions generates copious amounts of half-site products, the INs from equine infectious anemia virus (EIAV) and prototype foamy virus (PFV) promote predominantly concerted integration *in vitro* [30,64]. The reasons for these differences are currently unknown.

Although the IBD, the only region of LEDGF known to directly interact with IN, is sufficient to stabilize IN tetramers and to stimulate its 3′-processing activity [36], it is not sufficient to bolster strand transfer [43]. In fact, isolated LEDGF_IBD_ can competitively inhibit LEDGF-dependent strand transfer activity of HIV-1 IN [43]. Concordantly, over-expression of GFP-LEDGF_IBD_ fusions can potently suppress HIV-1 integration in human cells [60,62]. Using naked DNA targets, Turlure *et al*. [49] observed that a fragment spanning residues 226–530 of LEDGF retained approximately 50% of full-length LEDGF strand transfer stimulative activity. Stimulation of EIAV IN strand transfer activity required the presence of the DNA-binding AT hook region in the LEDGF construct (P.C., unpublished observations). Similar experiments using reconstituted polynucleosomes as target DNA revealed that the N-terminal PWWP domain is required for stimulation of HIV-1 integration into chromatinized DNA [100]. Furthermore, combining the IBD of LEDGF with heterologous chromatin binding domains, Meehan *et al*. [78] were able to create functional co-factors that rescued HIV-1 integration in LEDGF deficient cells. Thus, it seems likely that the effect of LEDGF on lentiviral IN activities is a combination of (*i*) enhancement of biologically-relevant multimerization, and, specifically pertaining to strand transfer, (*ii*) tethering of the PIC to target DNA. In addition, (*iii*) allosteric regulation of IN active site function by LEDGF cannot be ruled out at this point.

## 8. Concluding remarks, remaining questions and perspectives

Since its identification as an HIV-1 IN binding partner seven years ago, LEDGF and its role in retroviral replication has been subject to intense investigations. Despite initial controversy, the cellular protein has been validated as a *bona fide* co-factor of HIV-1 (and generally lentiviral) DNA integration. Recent studies revealed much about the functional and structural aspects of the IN-LEDGF interaction. Using RNA interference mediated knockdown and genetic knockout model systems several independent groups have demonstrated that the protein is important albeit not absolutely essential for HIV-1 integration, playing a major role in directing the virus into active transcription units of the host cell genome [59–61,72,73,101]. The most notable gap in our knowledge of LEDGF is its natural function in the cell. Even the intrinsic distribution of LEDGF along cellular chromatin is yet to be reported. The discovery of several LEDGF binding partners that in some but not all cases use LEDGF for chromatin tethering has done little to further our understanding of its native functions.

HIV DNA integration is an important target for antiretroviral drug discovery, and inhibition of the IN-LEDGF interaction is widely expected to produce a novel class of drugs [102,103]. Additionally, as a natural ligand of lentiviral INs, LEDGF has already served as a useful tool in structural biology of retroviral DNA integration [16,90]. We hope that using LEDGF-derived constructs will eventually allow crystallization and structure determination of the functional lentiviral PIC, which in turn would greatly stimulate the development of integrase inhibitors.

The IBD-NTD interface may also prove useful for exploitation in the design of safer gene therapy vectors. The recent success of creating artificial HIV-1 co-factors by linking alternative chromatin binding modules to the LEDGF IBD strongly advocates this possibility [78]. An extension of this work hypothesizes novel IBD fusion protein could target vector integration to safe genetic loci (see section 3, above). However, an obvious impediment to this strategy is the presence of endogenous LEDGF in target cells. This could potentially be overcome by using a mutant IN, unable to recognize endogenous cellular LEDGF, and a complementary IBD variant. Although the reverse-charge mutations provided a proof of principle that such a system could be designed [90], a more robust IN:IBD mutant pair needs to be developed for it to be applicable under conditions of endogenous LEDGF expression.

Recent genome-wide screening experiments have uncovered scores of cellular proteins required for HIV-1 infectivity [104–108]. Of these, transportin-SR2, shown to also bind IN *in vitro* [109], is already receiving attention of many laboratories, although a direct link between its interaction with IN and HIV-1 infection is yet to be established. The next few years should yield a wealth of functional and structural information about LEDGF, transportin-SR2, as well as novel potential IN co-factors [110], which will hopefully open new therapeutic possibilities.
